# Cerebral Venous Sinus Thrombosis is a Reversible Complication of Ulcerative Colitis

**DOI:** 10.7759/cureus.23099

**Published:** 2022-03-12

**Authors:** Hussain A Al Ghadeer, Sadeq A Alsalman, Jaafer Alobaid, Zainab I AlAbdi, Sultan S Aljereish, Shymaa Buhlaiqah, Maryam M Aljumah

**Affiliations:** 1 Paediatrics, Maternity and Children Hospital, AlAhsa, SAU; 2 Neurology, King Fahad Hospital-Hofuf, AlAhsa, SAU; 3 Radiology, King Fahad Hospital-Hofuf, AlAhsa, SAU; 4 Neurology, King Fahad Hospital-Hofuf, AlAhhsa, SAU; 5 Neurology, King Fahad Specialist Hospital, dammam, SAU; 6 Pediatrics, Maternity and Children Hospital, AlAhsa, SAU

**Keywords:** alahsa, saudi arabia, extraintestinal, ulcerative colitis, inflammatory bowel disease, thromboembolism, cerebral venous sinus thrombosis

## Abstract

Patients with inflammatory bowel disease (IBD) are at higher risk of venous thrombosis than the general population, with thromboembolism being a recognized extraintestinal manifestation. Although thrombotic events typically present as deep vein thrombosis and pulmonary embolism, other presentations are possible. Cerebral venous sinus thrombosis (CVST) is a relatively rare example associated with high morbidity and a mortality rate of 50% when misdiagnosed or the diagnosis is delayed. Despite this, CVST is a reversible complication with favorable outcomes when diagnosed early and treated appropriately. In this report, we present a case of cerebral sinus thrombosis in a 35-year-old female during a relapse of ulcerative colitis. During the relapse of ulcerative colitis, CVST manifested with a seizure, focal neurological deficit, and altered mental status. After blood workup, magnetic resonance imaging (MRI), and venography, the diagnosis of CVST was confirmed. We immediately started the patient on low-molecular-weight heparin, and during a six-month follow-up period, she made a full recovery with recanalization of the thrombosis on imaging. Despite CVST being a fatal complication of IBD, our report and data in the literature indicate that full remission is possible when it is correctly diagnosed and treated.

## Introduction

Inflammatory bowel disease (IBD) comprises Crohn’s disease, ulcerative colitis, and unclassified IBD. Among these, ulcerative colitis is diagnosed when the inflammation and ulceration of IBD are localized to the colon and rectum. The disease has an unknown etiology but is thought to result from the complex interaction of environmental and genetic factors [1,2], and although it affects any age group, it predominantly appears in women and people aged 15-30 years or 50-70 years [3]. Ulcerative colitis is characterized by local (intestinal) and systemic (extraintestinal) manifestations, with up to 50% of patients with IBD having extraintestinal manifestations in the skin, joints, bones, lungs, blood, eyes, kidneys, liver, and peripheral and central nervous systems [1,2]. Notably, IBD is considered a hypercoagulable state, with affected patients being at three to four times greater risk of developing thrombosis than the general population [4]. Deep venous thrombosis or pulmonary embolisms are the typical presentations, but rarer manifestations can also occur, such as cerebral venous sinus thrombosis (CVST) [5]. This report describes the presentation of a young woman who developed neurological symptoms during a relapse of ulcerative colitis. Early imaging revealed a CVST, and with prompt treatment, she achieved complete remission and recanalization.

## Case presentation

A 35-year-old Saudi female with known ulcerative colitis, diagnosed at the age of 20, presented to our emergency department with a two-week history of bloody diarrhea. She received regular gastroenterology follow-up, but although she was prescribed azathioprine and mesalamine, compliance was poor. Her current presentation was sudden and progressive, with a medium to large volume of hematochezia on five occasions, associated with intermittent central abdominal pain (severity, 7/10). In addition, she reported a single vomiting episode (food content only), dry cough, fatigue, drowsiness, and subjective fever for three days. She denied any history of headaches, visual disturbances, abnormal movement, confusion, joint pain, or changes in urine or skin. Her medical history also included depression, for which she received fluoxetine and regular psychiatric follow-up. Her history was otherwise negative for surgery, smoking, and a family history of IBD and genetic, hematological, or inherited disease.

Upon examination, she was conscious and alert with normal vital signs. Although she appeared pale and dehydrated and had mild diffuse abdominal tenderness, the examination revealed no other abnormality, including rebound tenderness or guarding. Laboratory investigation (Table 1) then revealed normocytic normochromic anemia, mild hyponatremia, and hypocalcemia with a low creatinine level, a normal liver profile, and elevated inflammatory markers. Stool samples were positive for occult blood and stool culture was positive for salmonella and shigella. Results were normal or negative for urinalysis, virology, and COVID-19 testing. Given these findings, we diagnosed an acute relapse of ulcerative colitis and admitted the patient to the hospital for treatment with hydrocortisone and infliximab, which we started after receiving a negative purified protein derivative test.

**Table 1:** Laboratory investigations

Laboratory investigations	Patient’s result	Reference level
Complete blood count		
White blood cells	1.67	10^9^/L (4–10)
Red blood cells	1.96	10^12^/L (3.8–4.8)
Mean corpuscular volume	93.4	81–99 FL
Hemoglobulin	5.9	12–15 g/dL
Platelets	196	10^9^/L (130–400)
Coagulation profile		
Prothrombin time (PT)	17.1	9.8–13.2 second
Partial thromboplastin time (PTT)	36.4	26–36 second
International normalized ratio (INR)	1.23	0.9–1.2%
D-dimer		
d-dimer	1.08	0–0.49 mcg/mL
Renal profile		
Urea	3.3	3.2–7.1 mmol/L
Creatinine	40	46–110 umol/L
Calcium total	1.96	2.1–2.5 mmol/L
Sodium serum	131	137–145 mmol/L
Potassium serum	4.1	3.5–5.1 mmol/L
Chloride serum	104	98–107 mmol/L
Liver profile		
Aspartate aminotransferase	11	15–46 U/L
Alanine aminotransferase	9	16–69 U/L
Alkaline phosphate	74	38–126 U/L
Total bilirubin	16.2	3–22 µmol/L
Direct bilirubin	3	0–5 µmol/L
Iron profile		
Iron	7.39	6–27 µmol/L
Total iron bending capacity	41	47–80 µmol/L
Inflammatory marker		
C-reactive protein	1.35	0–0.8 mg/dL
Erythrocyte sedimentation rate	108	0–15 mg/dL
Stool analysis		
Occult blood	Positive	
Culture	Positive for Salmonella and Shigella	
Urine analysis-profile		
All normal, no abnormality detected		
Thyroid function test		
FT3	1.55	2.8–7.1 pg/dL
Thyroid-stimulating hormone	1.27	0.5–5 mU/L
Antibodies		
Anti-tissue transglutaminase IGA	Negative	
Anti-endomysial antibodies	Negative	
Anti-SM	Negative	
Anti-SSB	Negative	
Virology profile		
Hepatitis b surface antigen	Negative	
Anti-HCV	Negative	
HIV antigen/antibodies	Negative	
Complement		
C3	77.4	
C4	24.7	

After two days of convalescence in the hospital, the patient had a generalized tonic-clonic seizure that lasted a few minutes and was followed by confusion, decreased consciousness, and left upper extremity weakness. Electroencephalography (EEG) showed a focal epileptic discharge with a secondary generalized seizure. We started valproic acid and transferred her to the intensive care unit. Neurological examination revealed a Glasgow Coma Scale (GCS) score of 12/15 and a Muscle Power Scale score of 4/5 with affected sensation in the left upper extremity. Other systemic examinations and repeat laboratory results were unremarkable except for normocytic normochromic anemia and elevated inflammatory markers.

**Figure 1:**
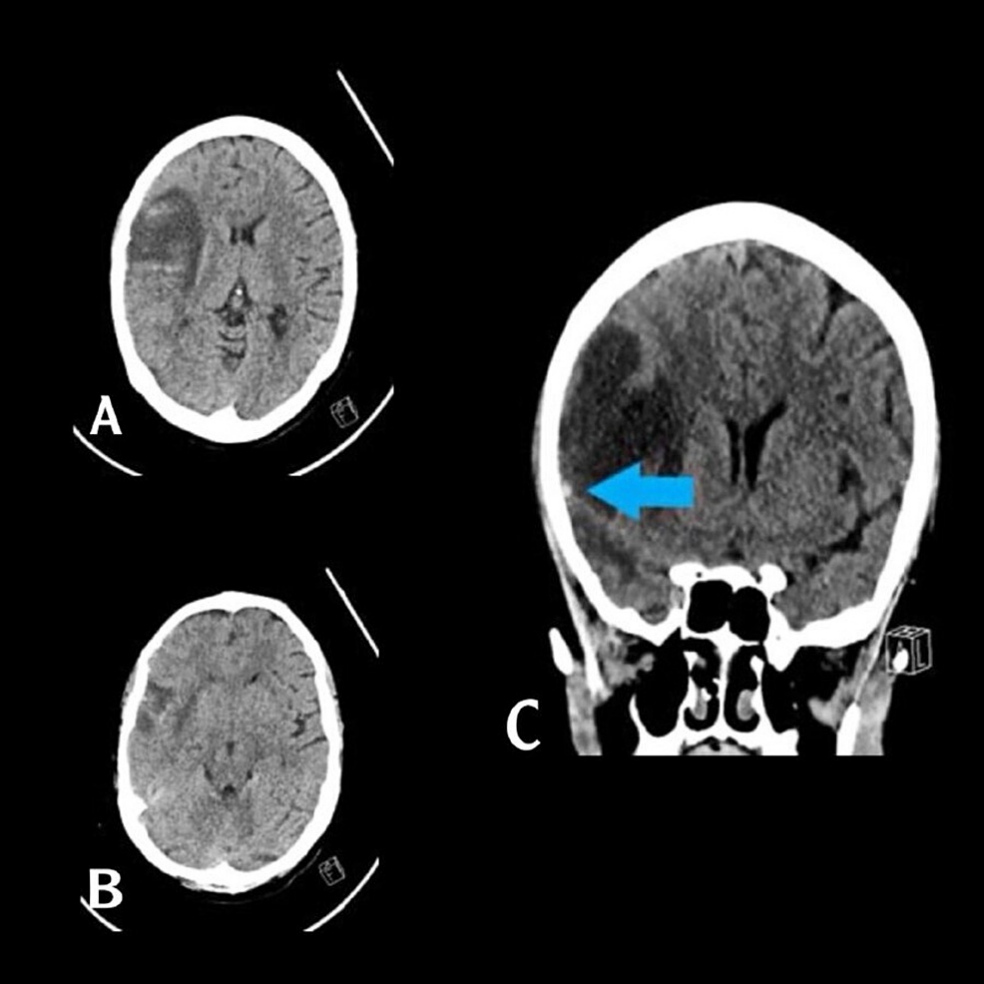
NECT axial (A,B) and coronal reformatted (C) show cortical and subcortical hypodensity with hyperdense foci (petechial hemorrhage) involving frontal, temporal, and insula on right side. Hyperdense cortical vein (red arrow).

**Figure 2:**
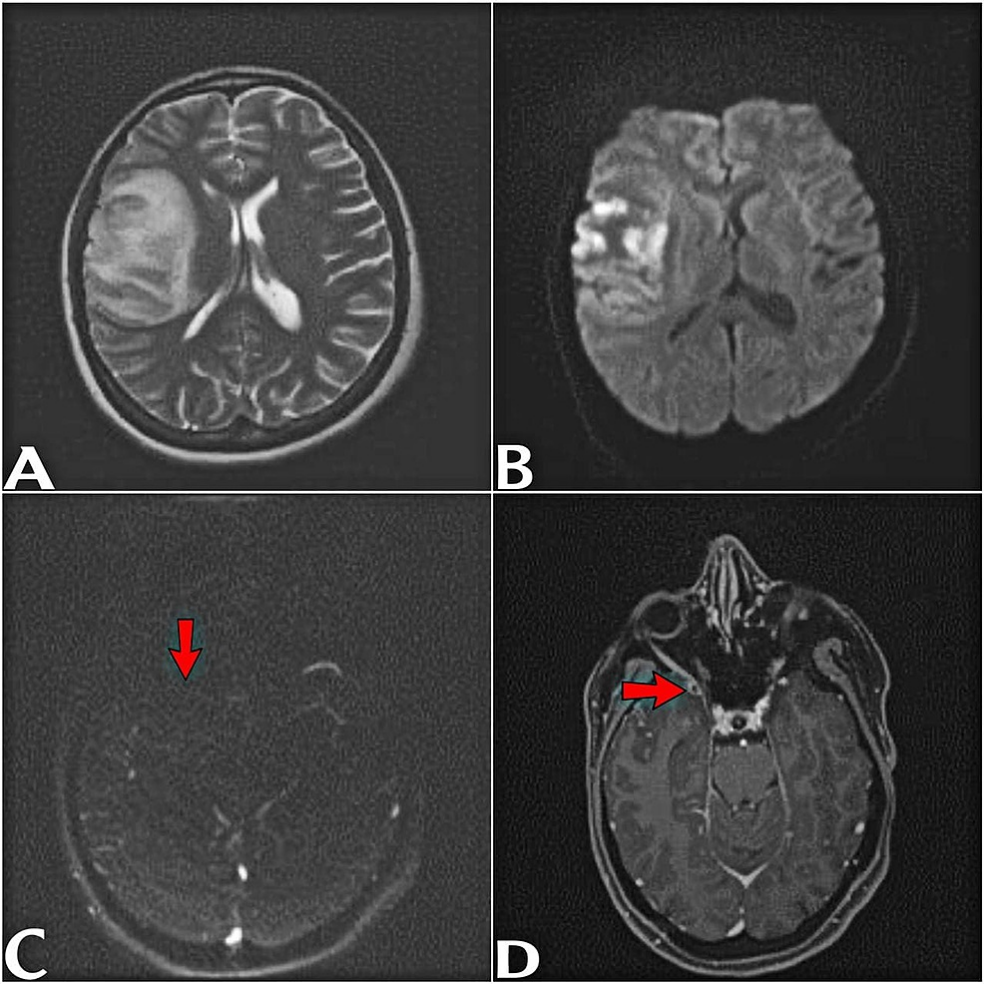
Axial T2WI (A) and DWI (B) in the same patient show hyperintensity and restricted diffusion in frontal lobe. Axial maximum intensity projection phase contrast MRV (C) shows absent flow in right sphenoparietal sinus (red arrow). Contrast-enhanced axial magnetization-prepared rapid gradient-echo (MP-RAGE), (D) shows filing defect in right sphenoparietal sinus (red arrow).

Initial assessment by computed tomography (CT), including angiography (CTA) and venography (CTV), showed an ischemic stroke from an infarction in the right middle cerebral artery territory (Figure 1A-1C). However, we suspected deep cortical venous thrombosis based on her history and presentation, so we requested urgent magnetic resonance imaging (MRI) and venography (MRV) with and without contrast. This revealed a thrombosis of the right-sided sphenoid parietal sinus and cortical veins, complicated by a venous infarction causing a mass (Figure 2A-2D).

**Figure 3:**
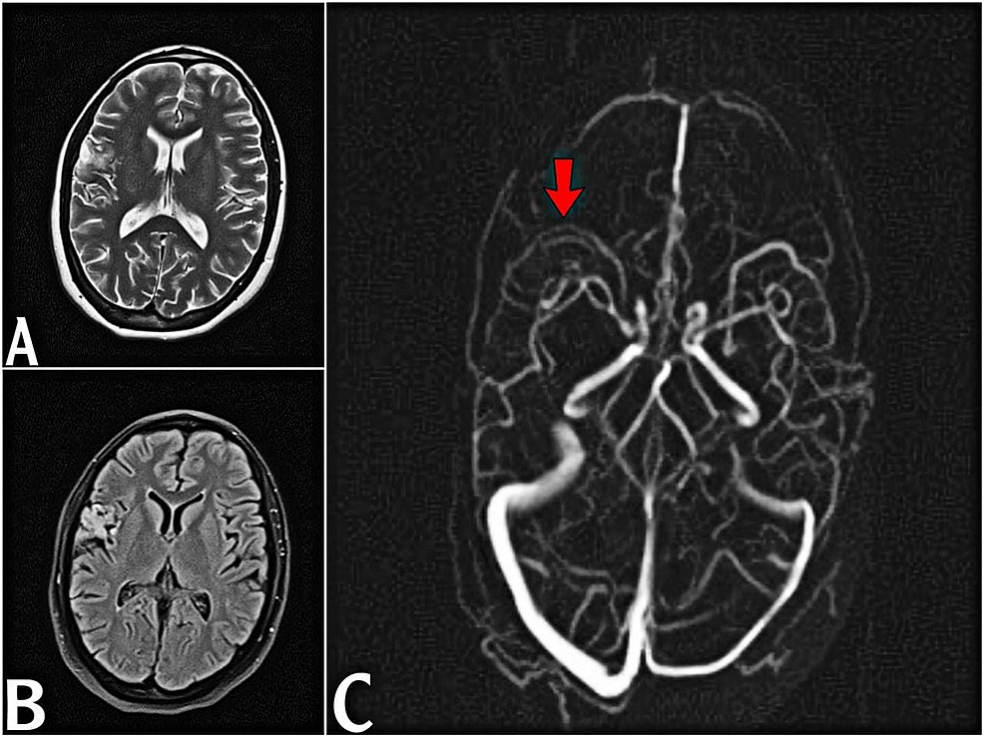
Axial T2 WI (A) and FLAIR WI (B) show mild volume loss with cystic changes and gliosis in right frontal lobe. Axial maximum intensity projection phase contrast magnetic resonance venography (C) shows recanalization of right sphenoparietal sinus (red arrow).

Given the clinical findings, we started therapeutic anticoagulation immediately. Over the next three days, the patient showed dramatic symptomatic improvement and a full recovery of her GCS and memory. All deficits are completely resolved over a two-week period (modified Rankin scale = 0). We, therefore, discharged him here with regular follow-up and the following oral medications: 2 mg warfarin per day for 6-12 months (optimized to the international normalized ratio), 1 g 5-aminosalicylic acid three times daily, 35 mg prednisone daily tapering by 5 mg weekly, 50 mg azathioprine daily, 40 mg fluoxetine daily, 50 mg lamotrigine twice daily, 20 mg omeprazole, 5 mg folic acid daily, 5000 IU vitamin D weekly, and 600 mg calcium carbonate twice daily. At six months, she had no residual neurological manifestations, and repeat MRI and MRV with and without contrast showed complete recanalization of the right-sided sphenoid parietal sinus and cortical veins (Figure 3A-3C). Currently, she is on a therapeutic dose of warfarin (9 mg). After seven months, a breakthrough seizure occurred, most likely due to poor compliance with antiepileptic medication; however, full remission was observed with no recurrence or residual neurological deficit.

## Discussion

CVST is rare compared with arterial thrombosis, with an incidence of 3-4/100,000 in adults and up to 7/100,000 in children [6]. Bargen et al. first reported an association between venous thromboembolism and IBD in 1936 [7]. In IBD, the incidence of CVST ranges from 0.5% to 6.7% [5], and it is relatively more common in ulcerative colitis than in Crohn’s disease [8]. Up to half of all patients with ulcerative colitis will develop extraintestinal manifestations, typically about 15 years after the initial diagnosis [1,2]. Patients with IBD are also at a fourfold increased risk of thrombosis compared with unaffected people [4]. In this report, our patient was diagnosed 15 years before developing CVST as an extraintestinal manifestation.

The exact mechanism of thromboembolism in IBD is poorly understood, though efforts have sought to explain the process. These include a hypercoagulable state due to elevated factor VIII and fibrinogen and by decreased antithrombin, protein S, and protein C; a hypofibrinolytic state due to elevated PAI‑1 and lipoprotein; platelet abnormalities and endothelial dysfunction due to increased von Willebrand factor; and immunological pathology due to antiphospholipid antibodies [9]. Thromboembolic events frequently appear during acute exacerbations or relapses of ulcerative colitis because the inflammatory reaction is associated with a hypercoagulable state that increases the risk of CVST [5]. In this report, we diagnosed CVST during a relapse of ulcerative colitis, supporting the proposition that disease activity is a predisposing factor for thromboembolic events.

The clinical manifestations of CVST vary from one patient to another, but patients classically present with a new-onset headache. Other manifestations include seizures, focal neurological deficits, confusion, altered mental status, and features of increased intracranial pressure [10]. New-onset neurological manifestations in patients with IBD should always raise suspicion of CVST to ensure that diagnosis and treatment occur early. The patient in this report developed a new-onset tonic-colonic seizure with a focal neurological deficit while recovering from a disease relapse, indicating the need to exclude CVST promptly.

The European Stroke Organization recommends measuring the D-dimer level prior to neuroimaging in cases of suspected CVST unless symptoms are limited to headaches and/or last longer than one week. However, this recommendation was rated as weak based on low-quality evidence [11], and it is generally agreed that CVST is best confirmed by neuroimaging studies. Head CT without contrast lacks sensitivity, but it remains an important tool because it can exclude hemorrhagic infarction that would otherwise contraindicate anticoagulation. Indeed, intracerebral hemorrhage is present in up to 30% of reported CVST cases and is associated with worse outcomes [12]. To confirm CVST, a patient should then undergo MRI and MRV with and without contrast to allow direct visualization of the thrombus [13]. The most common sites of cerebral thrombosis are the superior sagittal sinus and the lateral sinus [14]. In our patient, MRI and MRV provided accurate visualization of the CVST, showing thrombosis in the right-sided sphenoid parietal sinus and cortical veins that was complicated by venous infarction causing a mass.

The management of CVST in patients with IBD should focus on controlling the inflammation and dissolving the thrombus. Certain mediations used for IBD also inhibit platelet activation, such as 5-aminosalicylic acid, azathioprine, 6‑mercaptopurine, and infliximab, while steroids used in the active phase of ulcerative colitis also reduce intracerebral edema [15]. To dissolve the thrombus and prevent propagation, however, low-molecular-weight heparin should be used for anticoagulation [16]. Further attacks and complications can then be prevented by identifying those patients at high risk of abnormal hemostasis. In CVST, the American Heart Association and American Stroke Association recommend anticoagulation with a vitamin K antagonist for 3-6 months when there is a transient risk factor or 6-12 months when there is an identifiable risk factor [14]. The Canadian Association of Gastroenterology also recommends prophylactic anticoagulant use in patients hospitalized with severe relapses of IBD or treated as outpatients for moderate-to-severe relapses, especially when there is a history of thromboembolism or an identifiable risk factor [17]. CVST in IBD has no dedicated guidelines or recommendations, probably because of the limited number of case reports.

Kalita et al. reported that late-onset epileptic seizures recurred in up to 5.6% of patients with CVST [18]. Early follow-up with CTV or MRV should be performed for patients with progressive and persistent symptoms that suggest thrombus propagation despite medical treatment [19]. Later follow-up with CTV or MRV is recommended at 3-6 months to assess whether recanalization of the affected thrombus vein/sinus has occurred [20]. Although our patient developed a breakthrough seizure, we considered that this was probably due to poor compliance with therapy, given that we found no evidence of relapse. Follow-up MRI and MRV confirmed complete recanalization of the thrombosed vein or sinus, supporting the clinical observations that she had otherwise made a full recovery with no major recurrence or residual neurological deficit.

## Conclusions

CVST is a fatal complication of IBD that predominantly affects patients with ulcerative colitis. This report highlights the importance of considering this diagnosis in patients with IBD who present neurological manifestations of new-onset, especially when their underlying IBD is also relapsing. These patients rely on physicians' remaining vigilant and establishing an early diagnosis and proper treatment. Although laboratory and imaging studies have limitations, urgent D-dimer measurement and a CT scan without contrast are crucial to exclude differential diagnoses. Subsequently, MRI and MRV with or without contrast remain the gold standards for diagnosing CVST. A proper clinical approach with early diagnosis and adequate treatment is key to achieving the good outcomes seen in our patients. However, we require future studies to establish specific guidelines for the management of CVST in patients with IBD.
